# Glycerol Monolaurate Complex Improved Antioxidant, Anti-Inflammation, and Gut Microbiota Composition of Offspring in a Sow–Piglet Model

**DOI:** 10.3390/vetsci12010024

**Published:** 2025-01-07

**Authors:** Dan Li, Min Yang, Zhao Ma, Lianqiang Che, Bin Feng, Zhengfeng Fang, Shengyu Xu, Yong Zhuo, Jian Li, JiHhua Wang, Zhengfan Zhang, Zehui Wu, Tao Lin, De Wu, Yan Lin

**Affiliations:** 1 Key Laboratory of Animal Disease-Resistance Nutrition and Feed Science, Institute of Animal Nutrition, Sichuan Agricultural University, Chengdu 611130, China; xiaomila99@outlook.com (D.L.); danlidadan@163.com (Z.M.); che.lianqiang@sicau.edu.cn (L.C.); fengbin@sicau.edu.cn (B.F.); zfang@sicau.edu.cn (Z.F.); shengyu_x@hotmail.com (S.X.); zhuoyong@sicau.edu.cn (Y.Z.); 14109@sicau.edu.cn (J.L.); lt04161211@163.com (T.L.); wude@sicau.edu.cn (D.W.); 2 Pet Nutrition and Health Research Center, Chengdu Agricultural College, Chengdu 611130, China; yang040101@yeah.net; 3 Calid Biotech (Wuhan) Co., Ltd., Wuhan 430073, China; m18483617169@163.com; 4 Hubei Key Laboratory of Animal Nutrition and Feed Science, Wuhan Polytechnic University, Wuhan 430023, China; zhangzhengfan@163.com; 5 Sichuan Qiaozhu’er Breeding Co., Ltd., Neijiang 641100, China; wuzehui707@163.com

**Keywords:** sows, piglets, glycerol monolaurate complex, fecal microbial, reproductive

## Abstract

This study aimed to investigate the effects of maternal glycerol monolaurate complex (GML) and antibiotic (acetylisovaleryltylosin tartrate, ATLL) supplementation during late gestation and lactation on the reproductive performance of sows and the growth performance of piglets. In total, 64 pregnant sows were randomly divided into control, antibiotic, 0.1% GML, and 0.2% GML groups. The GML shortened their delivery interval and farrowing duration. The ATLL increased the level of malondialdehyde (MDA) in sows and piglets and enhanced glutathione peroxidase (GSH-Px) in piglets, while reducing the tumor necrosis factor-α (TNF-α) level in sows. The GML tended to increase milk protein in the colostrum and decreased the TNF-α of sows at lactation. Meanwhile, 0.2% GML increased the serum total superoxide dismutase (T-SOD) activity and interleukin-6 level in weaned piglets and decreased the TNF-α level in sows and weaned piglets. Furthermore, ATLL decreased the microbial diversity of sows, and GML tended to increase the microbial diversity of sows and piglets. The ATLL group had an increased relative abundance of Bacteroidota in weaned piglets. The GML decreased the relative abundance of Peptostreptococcales-Tissierellales, Proteobacteria, and the harmful bacteria *Romboutsia* in sows. Compared with the ATLL group, the 0.2% GML reduced the relative abundance of Bacteroidota in weaned piglets. Interestingly, both ATLL and GML supplementation decreased the relative abundance of harmful bacteria Peptostreptococcaceae in sows. Correlation analysis also found positive effects of ATLL and GML in anti-inflammatory and antioxidant aspects. In conclusion, GML enhanced reproductive and growth performance by improving antioxidant and anti-inflammatory status and maintaining intestinal flora balance, making it a promising alternative to ATLL in future applications.

## Simple Summary

Antibiotic additives have significant effects in relieving the stress of sow delivery and weaning piglets and improving disease resistance in animals. However, the abuse of antibiotics led to bacterial resistance. The purpose of this study was to investigate the effects of GML on the reproductive performance of sows and the growth performance of piglets in late gestation and lactation diets and to preliminarily explore its possible mechanism to replace antibiotics. In conclusion, 0.2% GML supplementation improved the reproductive performance of sows and the growth performance of piglets during late gestation and lactation by improving the antioxidant capacity, anti-inflammatory action, and gut microflora balance.

## 1. Introduction

The reproductive performance of sows is directly related to production efficiency and economic benefits of the swine industry [1]. With the dramatic changes in hormones and metabolism in the body, especially in the late gestation and early lactation stages, sows often suffer from fatigue, anorexia, constipation, long labor, weight loss, and other symptoms during lactation [2]. Antibiotic additives have significant effects in relieving the stress of sow delivery and weaning piglets and improving disease resistance in animals. However, the abuse of antibiotics has led to bacterial resistance [3], high antibiotic levels in livestock and poultry, as well as environmental pollution [4], and is a threat to human health [5,6]. European food unions and countries such as China have enforced a total ban on using antibiotics in livestock feed. This has undoubtedly increased the pressure on livestock farmers [7] and has prompted researchers to focus attention on biologically active natural alternatives to improve the reproductive performance of sows, along with the growth and health of piglets, by improving their intestinal and immune functions [5,8].

Glycerol monolaurate (C15H30O4, GML), a lipophilic medium-chain fatty acid ester naturally found in breast milk, palm oil, and coconut oil [9]. There are a variety of biological activities [10] and nutritional effects of GML [11]. More importantly, whereas most antibiotics have only a single bacterial target, GML has multiple cellular targets; therefore, use of GML does not easily result in development of resistance to drugs [10]. Studies have shown that GML can improve the daily gain of fattening cows [12], reduce the number of methanobacteria and protozoa in the rumen fluid of sheep [13], stabilize the intestinal flora, improve the growth performance of weaned piglets [14], and improve the feed conversion ratio (FCR), meat quality, intestinal morphology, and barrier function of broilers by enhancing animal immunity [15] and antioxidant balance, as well as promoting their intestinal flora health [16,17]. Additionally, GML is widely used in broilers to maintain the intestinal microecological balance and improve growth performance [5]. and in weaned piglets to increase beneficial intestinal bacteria and reduce diarrhea [18].

As bioactive plant compounds, essential oils contain mainly thymol and cinnamaldehyde (CIN). Research has demonstrated that thymol has beneficial effects on the growth of beneficial bacteria and inhibits the growth of potentially harmful bacteria, including Escherichia coli and Clostridium perfringens [19,20]. Also, CIN possesses bacteriostatic, anti-bacterial, and other functions [21]. It has been used to treat diabetes and diseases caused by Helicobacter pylori [22] by enhancing gastrointestinal peristalsis, maintaining intestinal microecological balance [23], and enhancing antioxidation [24], because its mechanism of action is similar to that of antibiotics [25]. Dietary supplementation with 0.75% GML and oregano essential oil can improve the intestinal structure and morphology, as well as growth performance of broiler chickens [5]. Wang et al. [26] found that dietary supplementation of 0.1% and 0.2% GML complex (mixture including GLM, CIN, and thymol) improved the growth performance of weaned piglets, and the addition of 0.2% GML complex also improved the antioxidant capacity. Zhao et al. [27] showed that maternal supplementation with 0.1% GML improved the composition of sow milk. These results indicate that GML supplementation not only promotes piglet growth and health but also benefits maternal milk composition and the intestinal health of offspring. Although studies on supplementing GML have increased in recent years, few have reported the effect of GML on sow reproductive performance. We hypothesized that GML has a positive effect on improving sow reproductive and piglet growth performance, and can be used as an alternative to natural antibiotics. The aim of this study was to investigate the effects of GML on sow reproductive performance, suckling piglet growth performance, blood indexes, antioxidant capacity, and fecal microorganisms, and to initially explore the possible mechanisms.

## 2. Materials and Methods

### 2.1. Animals and Diets

In total, 64 healthy pregnant Landrace × Yorkshire sows with similar parity and body condition (90 d of gestation, back fat 15.08 ± 3.41 mm) were divided into four treatment groups randomly: control (CON), antibiotic (0.3% acetylisovaleryltylosin tartrate, ATLL), 0.1% GML, and 0.2% GML, with 16 replications per group. The experiment lasted 45 d, from 90 d of gestation (G 90) to 21 d of lactation (L 21). The GML complex contained GML (800 g/kg) and essential oils CIN (54 g/kg) and thymol (6 g/kg), provided by CALID Biotechnology Co., Ltd. (Wuhan, China). Diets were according to the experimental design with reference to the nutritional requirements of sows in the NRC 2012 [28]. The nutritional parameters of the gestation and lactation diets is listed in Supplementary Table S1.

### 2.2. Management

During gestation, sows were individually housed in gestation stalls, with the temperature maintained at 20 °C. The sows received daily feed at 8:30 and 14:30, and were fed 2.8 kg/d during 90–110 d of gestation. At 110 d of gestation, sows were moved from the gestation stalls to farrowing rooms and were kept in individual farrowing crates thereafter with temperature maintained at 23 °C. The litter sizes were standardized to 10–12 piglets each at 24 h post-farrowing by cross-fostering within the same treatment. On the day of delivery, no feed was provided to the sows. Starting the day after delivery, they were given a lactation diet at 2.0 kg/d, which increased by 1 kg/d over the first 5 days, after which they had unrestricted access to the food. During lactation, sow’s milk was the sole source of nutrition for the suckling piglets. Both sows and piglets had continuous access to water throughout the study. All piglets were weaned at 21 days of lactation.

### 2.3. Growth Performance

The back fat thickness of the sows was measured at the P2 position using a B-mode ultrasonic device (Renco Lean Meater type 7, Minneapolis, MN, USA) at 90 d of gestation and 21 d of lactation.

Stool consistency and fecal morphology were recorded at 08:00 and 20:00 during gestation. Fecal scores were calculated according to Jing et al. [29], in which the scoring range was 0–4. A score of 3 or above was judged as constipated, and the constipation rate was calculated repeatedly.

### 2.4. Samples Collection

On the day of weaning, six piglets from each treatment group were randomly selected for collection of blood samples from the front cavity vein. At 1 and 21 d of lactation, six sows from each treatment group were randomly selected for collection of blood samples from the ear margin vein. Blood samples were centrifuged for 10 min at 3000 rpm at 4 °C after natural coagulation to obtain serum. Serum samples were immediately stored at −20 °C for further analysis.

At 20 d of lactation, six piglets, selected from different litters and weighing close to the average, were chosen per group, and feces were collected using rectal massage. The collection of feces of sows was carried out in the same way. Fecal samples were stored at −80 °C and tested for microbial diversity for sows and piglets.

Colostrum (10 mL) was collected from six sows per group from the 4th or 5th pair of nipples after the sow had delivered five piglets and was stored at −20 °C. Normal milk samples were collected from six sows from each group at 20 d of lactation and stored at −20 °C.

### 2.5. Determination

#### 2.5.1. Determination of Antioxidant Capacity and Cytokines

Total superoxide dismutase (T-SOD) and glutathione peroxidase (GSH-Px) activities, total antioxidant capacity (T-AOC), and malondialdehyde (MDA) content were determined using commercial kits (Jiancheng Bioengineering Institute, Nanjing, China). Cytokine levels—interleukin-1β (IL-1β), interleukin-6 (IL-6), interleukin-10 (IL-10), and tumor necrosis factor-α (TNF-α)—were measured using ELISA kits (Jiancheng Bioengineering Institute, Nanjing, China). The IgA content in colostrum was determined using a commercial kit (Jiancheng Bioengineering Institute, Nanjing, China).

#### 2.5.2. Determination of Milk Composition

The milk fat, lactose, milk protein, and total solid contents were determined using mid-infrared spectroscopy (Foss MilkoScan FT+, Fossomatic FC, Hillerød, Denmark).

#### 2.5.3. Analysis of Fecal Microbial Diversity

The number of microorganisms in feces of piglets and sows was determined using 16s rRNA. Bacterial genomic DNA was isolated from fecal samples of sows and piglets using a DNA extraction kit (Omega Biotek, Norcross, GA, USA) and quantified using Nanodrop (Thermo Fisher Scientific Inc., USA). The PCR amplification, purification, and product analysis were performed as described by Dai et al. (2022) [30]. The samples were analyzed by Majorbio Bio-Pharm Technology Corporation Ltd. (Shanghai, China). High-throughput sequencing was performed on a MiSeq sequencer using the Reagent Kit v3 according to the manufacturer’s instructions (Illumina Inc., San Diego, CA, USA) on the Genomic and Transcriptomic Platform (INRAE, Toulouse, France). Species composition was analyzed according to Le et al. [31].

## 3. Statistical Analysis

This trial was fully randomized with sow or litter size as the test unit, the sow model with diet treatment as the fixed effect, and estrus time as a random effect. The piglet model used sow diet treatment as a fixed effect and maternal difference as a random effect. All data were expressed as means, and data analysis was performed using one-way analysis of variance and statistical product and service solutions (SAS) statistical software (V9.4, SAS Institute Inc., Cary, NC, USA), followed by a generalized linear model. In the results, *p* < 0.01 was considered highly significant, *p* < 0.05 was significant, and 0.05 < *p* < 0.10 was a trend.

## 4. Results

### 4.1. Effects of GML on Back Fat and Fecal Scores of Sows

Maternal supplementation with GML had no significant effect on back fat loss or fecal scores of sows (*p* > 0.05; Table 1).

### 4.2. Effects of GML on Sow Reproductive Performances

Supplementation with GML shortened the delivery interval of sows (*p* < 0.05), and 0.1% GML shortened the farrowing duration of sows (*p* < 0.05), but had no significant effect on litter size, litter weight, or average body weight (*p* > 0.05; Table 2). Both ATLL and GML groups showed a trend of decreased incidence of lactating diarrhea in piglets (*p* < 0.1).

### 4.3. Effects of GML on Sow Feed Intake During Lactation

Compared to the ATLL group, 0.2% GML increased the feed intake for 1–7 d of lactation. Meanwhile, there was a trend for ATLL to reduce the feed intake of sows at 15–21 d and 1–21 d of lactation (*p* < 0.1) (Table 3).

### 4.4. Effects of GML on Composition of Colostrum and Normal Milk of Sows

Maternal supplementation with GML had no significant effects on fat, lactose, or total solids in the colostrum and milk of sows but tended to increase milk protein in colostrum (*p* < 0.1; Table 4). Moreover, 0.2% GML increased milk IgA content by 21.46% in colostrum and fat content by 9% in milk at 20 d of lactation.

### 4.5. Effects of GML on Antioxidant Capacity of Sows and Piglets

The ATLL increased the MDA levels of sows at 21 d of lactation and the serum GSH-Px levels in weaning piglets (*p* < 0.05; Table 5). The GML had no significant effect on the serum antioxidant indices of sows; however, 0.2% GML increased the serum T-SOD levels in weaning piglets (*p* < 0.05).

### 4.6. Effects of GML on Cytokine Secretion of Sows and Piglets

As shown in Table 6, ATLL and GML supplementation decreased the TNF-α of sows at 1 and 21 d of lactation (*p* < 0.05). The 0.1% GML increased the IL-10 of sows at 21 d of lactation (*p* < 0.05) and tended to increase serum IL-10 concentration of weaning piglets (*p* < 0.1). The 0.2% GML supplementation decreased the TNF-α level and increased the IL-6 concentration of weaning piglets (both *p* < 0.05).

### 4.7. Effects of GML on Gut Microbial Composition of Sows and Piglets

**Figure 1:**
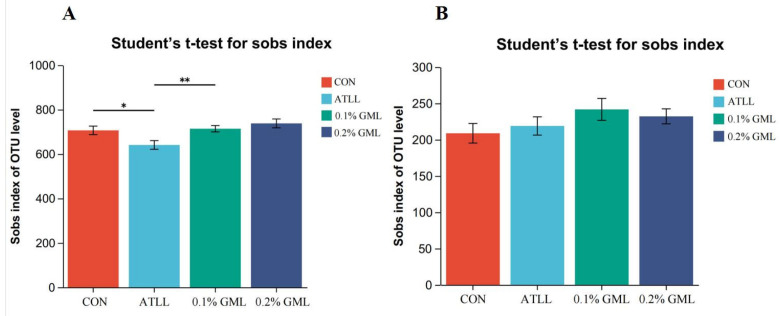
Analysis of microbial diversity of sows (**A**) and piglets (**B**). (**A**) Sows on day 20 of lactation. (**B**) Piglets on day of weaning. * *p* < 0.05, ** *p* < 0.01.

The Sobs index difference (Figure 1) showed that dietary supplementation with ATLL decreased the microbial diversity of sows compared with the CON group (*p* < 0.05; Figure 1A), whereas this increased in the 0.1% GML group (*p* < 0.01). However, this difference was not significant in piglets (*p* > 0.05; Figure 1B).

**Figure 2:**
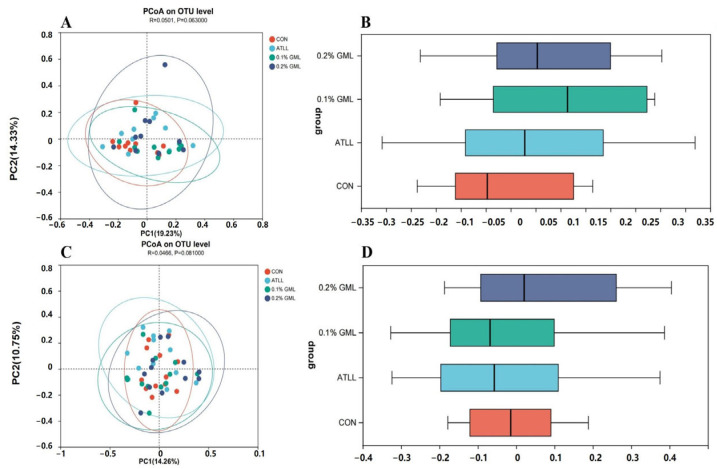
Effect of GML on intestinal microbial diversity of sows (**A**,**B**) and piglets (**C**,**D**). The different color legends represent different feeding feeds. CON, control group (basal diets), ATLL, antibiotic group (basal diets + 0.3% acetylisovaleryltylosin tartrate), 0.1% GML, glycerol monolaurate group (basal diets + 0.1% GML complex), 0.2% GML, 0.2% glycerol monolaurate group (basal diets + 0.2% GML complex).

Principal coordinate analysis based on Bray–Curtis distance showed a different trend in sows and piglets (*p* < 0.1; Figure 2). The 0.1% and 0.2% GML treatments both tended to increase the microbial diversity of sows and piglets. This indicated that GML addition contributed to increased gut microbial diversity.

**Figure 3:**
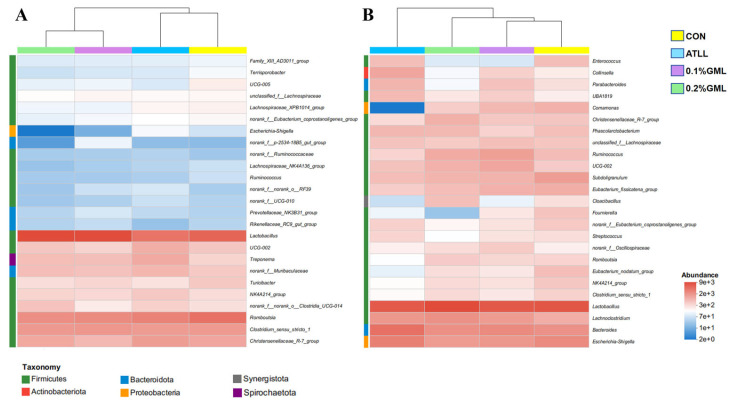
Bacterial community heat map analysis and sample cluster tree of genus level in sows (**A**) and piglets (**B**). CON, control group (basal diets), ATLL, antibiotic group (basal diets + 0.3% acetylisovaleryltylosin tartrate), 0.1% GML, glycerol monolaurate group (basal diets + 0.1% GML complex), 0.2% GML, 0.2% glycerol monolaurate group (basal diets + 0.2% GML complex). The abundance change of different species in the sample is displayed by the color gradient.

Bacterial community heat map analysis and sample cluster tree analysis at the genus level for the species composition of sows and piglets in the treatment groups are shown in Figure 3. At the genus level, composition of microbes in sows in the CON and ATLL groups was similar but differed from that in the GML group. The dominant genera in the piglet fecal microbiota in the ATLL group differed from those of other treatment groups.

**Figure 4:**
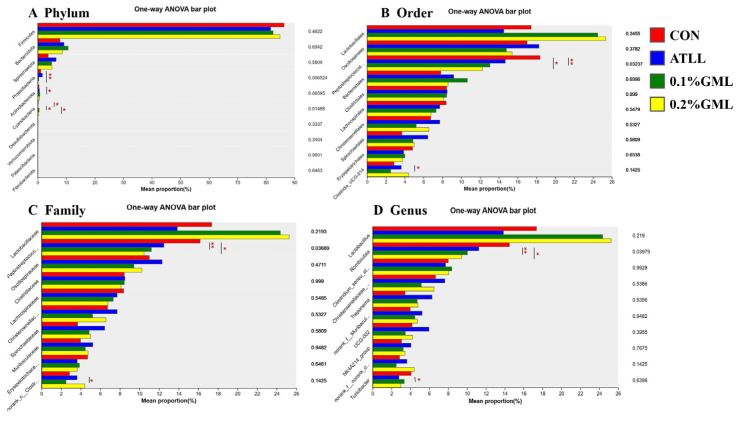
Analysis of group significant differences test among the top ten dominant phyla (**A**), orders (**B**), families (**C**), and genera (**D**) in fecal samples of sows. CON, control group (basal diets), ATLL, an-tibiotic group (basal diets + 0.3% acetylisovaleryltylosin tartrate), 0.1% GML, glycerol monolaurate group (basal diets + 0.1% GML complex), 0.2% GML, 0.2% glycerol monolaurate group (basal diets + 0.2% GML complex). The species names at the taxonomic level are shown on the *y* axis, while the *x* axis represents the average relative abundance. * *p* < 0.05, ** *p* < 0.01.

Significant differences among the top 10 dominant phylum (A), orders (B), families (C), and genera (D) in fecal samples of sows from different groups were determined (Figure 4). At the phylum level, addition of 0.1% and 0.2% GML decreased the relative abundance of Peptostreptococcales-Tissierellales (*p* < 0.05) and Proteobacteria (*p* < 0.01), respectively (Figure 4A). At the order level (Figure 4B), ATLL supplementation decreased the relative abundance of Lactobacillales (*p* > 0.1). However, the addition of 0.1% GML increased the relative abundance of Lactobacillales (*p* > 0.1)) and significantly decreased the relative abundance of Peptostreptococcales-Tissierellales (*p* < 0.01). At the family level, ATLL and GML supplementation both decreased the relative abundance of Peptostreptococcaceae (*p* < 0.05; Figure 4C). At the genus level, GML supplementation decreased the relative abundance of *Romboutsia* (*p* < 0.05; Figure 4D).

**Figure 5:**
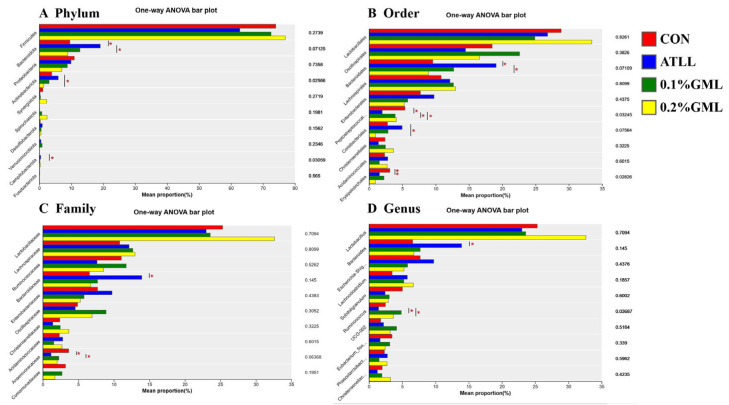
Analysis of group significant differences test among the top ten dominant phyla (**A**), orders (**B**), families (**C**), and genera (**D**) in fecal samples of piglets. CON, control group (basal diets), ATLL, antibiotic group (basal diets + 0.3% acetylisovaleryltylosin tartrate), 0.1% GML, glycerol monolaurate group (basal diets + 0.1% GML complex), 0.2% GML, 0.2% glycerol monolaurate group (basal diets + 0.2% GML complex). The *y* axis shows species names at the taxonomic level, while the *x* axis displays the average relative abundance of the species. * *p* < 0.05 and ** *p* < 0.01.

Changes in the gut microbiota of mothers may affect the microbial composition of their offspring. As shown in Figure 5A, at the phylum level, the changes in microorganisms in piglets were similar to those in sows. The ATLL group significantly increased relative abundance of Bacteroidota (*p* < 0.05). Treatment with 0.2% GML significantly decreased the relative abundance of Actinobacteria (*p* < 0.05). It is worth mentioning that compared with the ATLL group, the 0.2% GML treatment significantly reduced the relative abundance of Bacteroidota (*p* < 0.05). At the order level (Figure 5B), the ATLL group showed a significant increase in the relative abundance of Bacteroidales (*p* < 0.05), however, the 0.2% GML treatment significantly reduced the relative abundance of Bacteroidales (*p* = 0.018). At the family level (Figure 5C), the ATLL treatment significantly increased the relative abundance of Bacteroidaceae (*p* < 0.05). At the genus level (Figure 5D), the ATLL treatment significantly increased the relative abundance of *Bacteroides* (*p* < 0.05). whereas GML supplementation significantly increased the relative abundance of *Ruminococcus* (*p* < 0.05), compared with the ATLL group.

**Figure 6:**
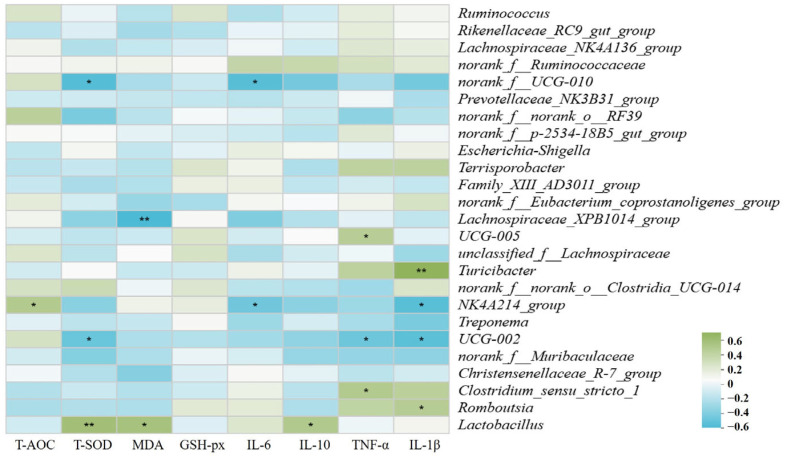
Heat map of correlation analysis between microbial composition and the antioxidant/inflammatory cytokine levels in sows at the genus level. The Spearman’s correlation coefficient r, ranging from −1 to 1, represents the value shown on the middle heat map. A value of R < 0 indicates a negative correlation, while R > 0 indicates a positive correlation. * *p* < 0.05 and ** *p* < 0.01.

The Spearman correlation analysis between microbial composition and the antioxidant/inflammatory cytokine levels in sows is shown in Figure 6. This analysis revealed distinct correlations between bacterial genera and the body’s antioxidant and inflammatory cytokine levels, highlighting the intricate role these bacteria play in regulating both antioxidant activity and inflammation. Specifically, the relative abundance of *Lactobacillus* was positively correlated with levels of T-SOD and IL-10 (R > 0.4, *p* < 0.05). In contrast, the relative abundance of *UCG-002* showed a negative correlation with T-SOD and TNF-α (R < −0.4, *p* < 0.05). Similarly, *norank_f_UCG-010* was negatively correlated with T-SOD, IL-6, and IL-1β (R < −0.4, *p* < 0.05). Finally, the *NK4A214_group* demonstrated a positive correlation with T-AOC (R > 0.4, *p* < 0.05) while being negatively correlated with IL-6 and IL-1β (R < −0.4, *p* < 0.05).

## 5. Discussion

Long labor can increase the risk of hypoxia and death in piglets, especially for the lastborn or lightest piglets [32]. In the present study, GML supplementation shortened the delivery interval and farrowing duration of sows. The addition of GML increased the weaning litter weight of piglets. Further analysis showed that 0.2% GML increased the IgA in colostrum and thus reduced diarrhea incidence in piglets during lactation. Changes in milk composition are conductive to piglet growth [33]. Li et al. [34] found that the addition of α -GML to the diet improved the milk immunoglobulin of sows, thus improving the health of piglets, and the IgA and IgG levels increased in sows along with the diet α -GML. GML maintained sow body condition and increased weight gain in piglets [35], while maternal 0.1% GML supplementation significantly increased fat and protein in colostrum and normal milk of sows [27]. However, GML can enhance the performance of weaned piglets by stabilizing the intestinal microbiota [14]. The concentrations of polyunsaturated and monounsaturated fatty acids in chicken egg yolk were increased with the addition of 0.3 g/kg GML. [16]. It was reported that, as a single medium-chain fatty acid glycerate, GML could be absorbed quickly and directly by piglets without emulsification, thereby reducing energy consumption [36]. However, medium-chain fatty acids in sows found higher colostrum protein concentrations [37]. Supplementation of GML in sows resulted in piglets with improved apparent total tract digestibility of dry matter, crude protein, and ether extract [34], implying that GML supplementation may benefit nutrient absorption by changing milk composition and piglet growth.

Before and after delivery, sows usually experience severe inflammatory reactions and oxidative stress that damage tissues and cells [38]; at the same time, this also damages the health of piglets [39]. Serum SOD and GSH-Px, which are antioxidant enzymes, can remove excess free radicals and play an important role in maintaining the oxidation balance in the body [40], and MDA as an oxidative product is considered a marker of oxidative stress [41]. In this study, addition of antibiotics increased the MDA level in sows in late lactation. Many studies have shown that antibiotic treatment induces the oxidative damage commonly caused by bacteria, leading to the production of reactive oxygen species, and causing oxidative stress that leads to cell death [42]. In contrast, ATLL and 0.2% GML contribute to activating the antioxidant enzyme system in weaned piglets, thus protecting them from excessive oxidative damage. Similar findings were reported in poultry [43].

Previous studies have indicated that modulation of the intestinal microbiota by antibiotics may suppress the immune response and alleviate inflammation [44,45]. In mouse experiments, the use of antibiotics can reduce the levels of the pro-inflammatory cytokine TNF-α [46,47]. Furthermore, in this study, application of ATLL and GML both significantly decreased the secretion of the pro-inflammatory cytokine TNF-α in sows, suggesting that GML may have a similar effect to ATLL in mitigating inflammatory responses. It has been shown that GML can effectively regulate pro-inflammatory factors, and inflammatory and immune responses by inhibiting the expression of inflammatory factors, chemokines, and cytokines [17,48]. Additionally, it has been found that GML can inhibit pro-inflammatory factors such as IL-2, IFN-γ, TNF-α, and IL-10 in a dose-dependent manner [15]. The levels of TNF-α and IL-10 in lactating sows and weaned piglets supplemented with different doses of GML corroborated these findings. However, we found in piglets given 0.1% and 0.2% GML that the low doses reduced the IL-6 level, but this was not dose-dependent. We speculate that this discrepancy may be related to the pleiotropic effects of IL-6, which can induce B cell differentiation and immunoglobulin production to mitigate inflammation [49], although the specific mechanism remains unclear. The above conclusions suggest that both ATLL and GML can effectively regulate inflammation and immune responses, and that maternal GML supplementation has more beneficial effects on offspring after birth than ATLL.

The intestinal microbiota is widely regarded as a key regulator of host physiology [50], oxidative stress [51], immune homeostasis [52], and reproductive performance [53]). Additionally, mother-to-infant microbial transmission has been observed in humans [54] and pigs [47]. Previous studies indicated that the use of antibiotics has a negative impact on the gut microbiota, leading to a reduction in species diversity, thus affecting stability of the gut microbiota and adversely affecting the host’s gut health [55]. In contrast, research has demonstrated that GML helps maintain the integrity of the intestinal barrier function, promoting gut health [16,17]. Additionally, GML can improve the growth performance of weaned piglets and stabilize the gut microbiota [14]. The results of our study are consistent with previous findings, indicating that the use of antibiotics alters the composition of the gut microbiota, leading to a decrease in microbial diversity, while GML helps increase microbial diversity.

Gut microbes can affect the antioxidant response of the gut, and once the gut microbiota is unbalanced, it will destroy the intestinal barrier, increase the oxidative damage and reduce the antioxidant capacity [51]. It was found that 18 microbial genera were associated with antioxidant capacity of growing pigs [56]. Probiotics can play a positive role in regulating the oxidative stress response by scavenging free radicals and enhancing the activity of antioxidant enzymes [57,58]. In addition to participating in antioxidant responses, microorganisms also reduce intestinal inflammation by promoting the expression of anti-inflammatory factors and inhibiting the production of proinflammatory cytokines [59,60]. Tang et al. [61] observed that the relative abundance of *Romboutsia* and *Peptostreptococcaceae* was negatively correlated with immune characteristics. Wang et al. [47] found that *Bacteroides* play an important role in immunity and anti-inflammation. Based on the correlation analysis between microbiota and antioxidants, our study suggests that *Lachnospiraceae_XPB1014_group*, *NK4A214_group*, *Lactobacillus,* and *UCG-002* may play a positive role in producing antioxidant and anti-inflammation effects. Furthermore, research suggests that antibiotics can influence the host’s inflammatory immune response by altering the microbial community [62,63]. GML can upregulate the favorable microbial abundance and reduce the occurrence of inflammation [17]. Maternal GML supplementation enhanced intestinal oxidative stability and barrier function in offspring and attenuated intestinal inflammatory responses in offspring [64]. Our study showed that adding antibiotics and GML improved the body immunity by adjusting the abundance of microorganisms associated with immunity. Meanwhile, the correlation analysis also found the positive effects of ATLL and GML in anti-inflammatory and antioxidant aspects. These findings strongly support the hypothesis that the antibiotic and GML groups may reduce inflammation by improving composition of the intestinal microbiota.

Energy intake was negatively correlated with an increase in Bacteroidetes’ relative abundance [65]. Our study showed that the relative abundance of Bacteroidetes increased in the antibiotic group, but decreased in the 0.2% GML group. In this 0.2% GML group, host growth increased by promoting energy intake, while antibiotics had the opposite effect. This may be because GML is conducive to reducing pH in the intestinal tract and this has a strong bactericidal effect [66], which can reduce the relative abundance of *Escherichia coli* [67] in the intestinal tract and increase the number of beneficial bacteria such as *Lactobacillus* [68]. Additionally, the effect of GML, which encourages the competitive growth of good bacteria but is detrimental to proliferation of pathogenic bacteria, may be associated with improved intestinal microbiota structure in sows and piglets. Studies have shown that 450 mg/kg GML can increase beneficial microorganisms, such as intestinal *Bifidobacteria* and *Lactobacillus* [69]. Consequently, the improved reproductive performance of sows and the growth performance of piglets were also attributed to the improved intestinal microbiota due to GML. This finding is important for understanding the potential role of antibiotic alternatives in maintaining gut health and their application in the livestock industry.

Overall, GML presents numerous advantages over traditional antibiotics, such as reduced oxidative stress, improved immune function, enhanced gut health, better growth performance, and beneficial effects on reproductive performance. However, despite these promising benefits, its full potential has yet to be fully explored. Further research is essential to better understand the underlying mechanisms of GML’s effects and to optimize its application in livestock production.

## 6. Conclusions

Maternal ATLL supplementation increased maternal oxidative stress responses by interfering with gut microbial composition while enhancing autoimmunity through synergistic effects. Dietary supplementation with GML in late-gestation and lactating sows can enhance reproductive performance by reducing the sow–piglet inflammatory response and increasing the piglets’ antioxidant capacity. However, maternal GML supplementation improved sow–piglet gut microbial diversity and balance through maternal microbial transmission, thereby improving growth of piglets and reducing the rate of piglet diarrhea by reducing the sow–piglet inflammatory response.

## Figures and Tables

**Table 1:** Effects of GML complex on back fat loss and fecal score of sows.

Items	CON	ATLL	0.1% GML	0.2% GML	SEM	*p*-Value
Sow Back Fat Thickness, mm						
Day 90 of Gestation	14.81	15.06	15.07	15.38	0.43	0.98
Farrowing, within 24h	16.00	15.30	15.14	13.80	0.36	0.56
Day 21 of Lactation	14.64	15.33	14.43	13.03	0.35	0.69
Total Back Fat Loss	−1.47	−1.08	−1.14	−1.68	0.22	0.89
Fecal Scores	1.66	1.42	1.41	1.58	0.08	0.64

Data are presented as mean (sows: n = 16). CON, control group (basal diets), ATLL, antibiotic group (basal diets + 0.3% acetylisovaleryltylosin tartrate), 0.1% GML, glycerol monolaurate group (basal diets + 0.1% GML complex), 0.2% GML, 0.2% glycerol monolaurate group (basal diets + 0.2% GML complex).

**Table 2:** Effects of GML complex on delivery performance of sows.

Items	CON	ATLL	0.1% GML	0.2% GML	SEM	*p*-Value
Farrowing Duration, min	195.4 ^b^	154 ^ab^	120.53 ^a^	185.81 ^b^	9.26	0.01
Delivery Interval, min	23.6 ^b^	19.59 ^ab^	13.1 ^a^	12.75 ^a^	1.48	0.02
Litter Size, n						
Total Born	11.69	13.13	12.80	13.73	0.46	0.29
Born Alive	11.00	11.75	11.73	12.57	0.43	0.45
Weaning	9.28	10.81	10.09	11.25	0.32	0.60
Litter Weight, kg						
Day 0 of Birth	15.18	14.82	14.86	17.11	0.56	0.24
Day of Weaning	62.82	59.42	60.11	66.73	1.65	0.33
Average Pig Body Weight, kg						
Day 0 of Birth	1.39	1.30	1.36	1.31	0.04	0.37
Day of Weaning	6.29	6.09	6.39	6.50	0.13	0.46
Fecal Score of Piglets	0.57	0.56	0.52	0.36	0.01	0.22
Diarrhea Rate of Piglets, %	1.46	1.14	0.92	0.84	0.32	0.08

Values are presented as mean (sows: n = 16). CON, control group (basal diets), ATLL, antibiotic group (basal diets + 0.3% acetylisovaleryltylosin tartrate), 0.1% GML, glycerol monolaurate group (basal diets + 0.1% GML complex), 0.2% GML, 0.2% glycerol monolaurate group (basal diets + 0.2% GML complex).

**Table 3:** Effects of GML complex on feed intake of sows during lactating.

Items	CON	ATLL	0.1% GML	0.2% GML	SEM	*p*-Value
G90-D0, kg	2.88	2.87	2.84	2.86	0.01	0.21
L1-L7, kg	4.1 ^ab^	3.9 ^a^	4.07 ^ab^	4.19 ^b^	0.03	0.03
L8-L14, kg	6.27	5.79	5.9	6.19	0.09	0.2
L15-L21, kg	6.34	5.8	5.88	6.19	0.08	0.06
L1-L21, kg	5.57	5.16	5.27	5.51	0.06	0.07

Abbreviations: G90 = day 90 of gestation; D0 = day 0 of delivery; L1 = day 1 of lactation. Data are presented as mean (sows: n = 16). CON, control group (basal diets), ATLL, antibiotic group (basal diets + 0.3% acetylisovaleryltylosin tartrate), 0.1% GML, glycerol monolaurate group (basal diets + 0.1% GML complex), 0.2% GML, 0.2% glycerol monolaurate group (basal diets + 0.2% GML complex.

**Table 4:** Effects of GML complex on the composition of colostrum and normal milk of sows.

Items	CON	ATLL	0.1% GML	0.2% GML	SEM	*p*-Value
Colostrum						
Fat, %	6.65	7.75	7.33	6.92	0.31	0.17
Protein, %	14.55	16.29	16.99	17.59	0.57	0.08
Lactose, %	2.75	2.65	2.40	2.48	0.08	0.40
Total Solids, %	28.56	31.75	30.99	30.94	0.76	0.33
IgA, mg/ml	4.80	3.26	4.49	5.83	0.56	0.46
Milk on Day 20 of Lactation						
Fat, %	6.65	6.86	6.99	7.25	0.29	0.22
Protein, %	5.02	5.08	5.05	5.09	0.12	0.35
Lactose, %	5.52	5.25	5.25	5.22	0.07	0.65
Total Solids, %	19.32	20.60	19.93	20.10	0.33	0.54

Data are presented as mean (sows: n = 6). CON, control group (basal diets), ATLL, antibiotic group (basal diets + 0.3% acetylisovaleryltylosin tartrate), 0.1% GML, glycerol monolaurate group (basal diets + 0.1% GML complex), 0.2% GML, 0.2% glycerol monolaurate group (basal diets + 0.2% GML complex).

**Table 5:** Effects of GML complex on serum antioxidant indices of sows and piglets.

Items	CON	ATLL	0.1% GML	0.2% GML	SEM	*p*-Value
Day 1 of lactation						
T-AOC, U/mL	2.89	2.73	2.73	2.67	0.10	0.95
T-SOD, U/mL	78.02	75.18	74.37	76.23	0.76	0.38
MDA, nmol/mL	4.94	4.15	5.09	5.20	0.38	0.48
GSH-Px, U/mL	785.53	739.42	779.27	676.22	19.70	0.18
Day 21 of lactation						
T-AOC, U/mL	3.01	3.38	3.29	3.02	0.14	0.73
T-SOD, U/mL	54.27	50.38	54.28	53.78	1.41	0.75
MDA, nmol/mL	3.94 ^a^	5.13 ^b^	4.59 ^ab^	4.55 ^ab^	0.19	0.04
GSH-Px, U/mL	667.60	558.86	660.51	567.36	30.10	0.58
Weaning piglets						
T-AOC, U/mL	3.38	3.40	4.07	5.154	0.14	0.43
T-SOD, U/mL	23.73 ^a^	38.25 ^ab^	39.80 ^ab^	49.53 ^b^	3.08	0.02
MDA, nmol/mL	7.31	8.315	8.07	7.09	0.41	0.27
GSH-Px, U/mL	140.73 ^a^	210.70 ^b^	145.86 ^a^	187.91 ^ab^	10.29	0.03

^a,b^ Values in the same row with different small letter superscripts mean significant difference (*p* < 0.05).T-AOC, total antioxidant capacity; T-SOD, total peroxide dismutase; MDA, malondialdehyde; GSH-Px, glutathione peroxidase. Data are presented as mean (sows: n = 6; piglets: n = 6). CON, control group (basal diets), ATLL, antibiotic group (basal diets + 0.3% acetylisovaleryltylosin tartrate), 0.1% GML, glycerol monolaurate group (basal diets + 0.1% GML complex), 0.2% GML, 0.2% glycerol monolaurate group (basal diets + 0.2% GML complex).

**Table 6:** Effects of GML complex on cytokines and immunoglobulins in sows and piglets.

Items	CON	ATLL	0.1% GML	0.2% GML	SEM	*p*-Value
Day 1 of lactation						
IL-1β, ng/L	202.18	188.38	186.77	199.66	6.88	0.76
TNF-α, ng/L	264.53 ^b^	203.27 ^a^	198.09 ^a^	220.67 ^a^	16.73	0.05
IL-6, ng/L	61.94	63.10	56.69	48.49	3.45	0.45
IL-10, ng/L	124.73	122.59	123.50	123.69	5.24	1.00
Day 21 of lactation						
IL-1β, ng/L	181.64	185.91	172.90	179.62	10.07	0.98
TNF-α, ng/L	367.28 ^c^	322.02 ^b^	338.63 ^b^	303.53 ^a^	34.14	0.05
IL-6, ng/L	60.69	58.77	68.40	59.23	4.03	0.84
IL-10, ng/L	124.19 ^a^	129.44 ^a^	154.39 ^b^	140.41 ^ab^	6.54	0.05
Weaning piglets						
IL-1β, ng/L	68.58	85.12	77.80	64.51	4.16	0.31
TNF-α, ng/L	115.54 ^b^	99.191 ^b^	110.35 ^b^	76.76 ^a^	12.45	0.01
IL-6, ng/L	40.85 ^a^	57.70 ^ab^	29.35 ^a^	85.64 ^b^	6.65	0.01
IL-10, ng/L	66.76	58.98	77.42	51.39	4.23	0.08

^a,b,c^ Values in the same row with different small letter superscripts mean significant difference (*p* < 0.05). Data are presented as mean (sows: n = 6; piglets: n = 6). CON, control group (basal diets), ATLL, antibiotic group (basal diets + 0.3% acetylisovaleryltylosin tartrate), 0.1% GML, glycerol monolaurate group (basal diets + 0.1% GML complex), 0.2% GML, 0.2% glycerol monolaurate group (basal diets + 0.2% GML complex).

## Data Availability

The datasets presented in this study can be found in online repositories. The names of the repository/repositories and accession number(s) can be found at https://www.ncbi.nlm.nih.gov/ accessed on 20 December 2024, PRJNA913793.

## Supplementary Materials

The following supporting information can be downloaded at https://www.mdpi.com/article/10.3390/vetsci12010024/s1, Table S1: The nutritional parameters of the gestation and lactation diet [70].
