# Supplementary material for: Glycerol Monolaurate Complex Improved Antioxidant, Anti-Inflammation, and Gut Microbiota Composition of Offspring in a Sow–Piglet Model
Source: Vet Sci. 2025 Jan 7;12(1):24. doi: 10.3390/vetsci12010024 (PMC11769162; doi:10.3390/vetsci12010024)
Supplement: Supplementary file 1 [file vetsci-12-00024-s001.zip › vetsci-3346688-supplementary.pdf]

Supplementary Table S1

The nutritional parameters of the gestation and lactation diet[1]

Items	Gestation	Lactation
Net Energy, kcal/kg	2300	2650
Crude Protein, %	13	16
Crude Fat, %	4	5.55
Total Lysine, %	0.75	1.15
Ca, %	0.70	0.90
P, %	0.60	0.65

References

1. Lin, Y., D. Li, Z. Ma, L. Che, B. Feng, Z. Fang, S. Xu, Y. Zhuo, J. Li, L. Hua, D. Wu, J. Zhang, and Y. Wang. "Maternal Tributyrin Supplementation in Late Pregnancy and Lactation Improves Offspring Immunity, Gut Microbiota, and Diarrhea Rate in a Sow Model." *Front Microbiol* 14 (2023): 1142174.
